# The Effects of Anger Management on Workers: A Questionnaire Survey of Organizational Dysfunctional Behavior and Withdrawal from Interpersonal Relationships in the Workplace

**DOI:** 10.3390/bs15020157

**Published:** 2025-02-01

**Authors:** Ryoichi Semba

**Affiliations:** Department of Management, Kyoto Tachibana University, Kyoto 607-8175, Japan; semba@tachibana-u.ac.jp

**Keywords:** aggression, anger, anger management, organizational dysfunctional behavior, withdrawal from interpersonal relationships

## Abstract

The voluntary behaviors of individuals that negatively impact the organization and its members should be addressed. According to the threatened egotism model, it is possible to curb such behaviors by effectively managing negative emotions. One such management strategy is anger management. Recently, some organizations have been implementing anger management programs, but their effect on behavior has not been verified. This study focused on organizational dysfunctional behavior and interpersonal withdrawal and attempted to examine the effects of anger management on these behaviors using a pre–post-test design. An anger management program and questionnaires before and after were administered to 92 workers (28 men and 64 women). The questionnaire included the Sociability Scale, which measures disengagement from interpersonal relationships, and the Organizational Dysfunctional Behavior Scale. A comparison of scale scores before and after the program revealed a significant decrease in the “Criticism of Others” on the Organizational Dysfunctional Behavior Scale. Furthermore, a similar analysis by sex and age revealed a significant decrease in “Criticism of Others” among women and workers under 46 years of age. These results indicate that anger management is not effective for reducing interpersonal withdrawal but may instead be effective for reducing criticism of others among women and young workers.

## 1. Introduction

In organizations, there are supervisors who are unable to scold their subordinates or workers and find it difficult to point out necessary issues because they are uncomfortable with interpersonal relationships (Hyugano & Oguchi, 2002). There are also workers who engage in aggressive behavior, such as blaming or bad-mouthing others (Semba & Haraguchi, 2014). In order for an organization to survive, productivity must be increased, which requires the effective and mutual communication of intentions. In particular, when leaders act as managers, reconciling the personal desires of their subordinates with the goals of the organization is important (Barnard, 1938; Chae, 2015). However, the presence of such interpersonal problems prevents the effective communication of intentions, and as a result, it is predicted that the organization’s productivity will not increase; in the worst case, the organization’s survival could be jeopardized (Booth & Mann, 2005).

Aggressive behavior in organizations is considered voluntary deviant behavior by individuals and comprises a wide range of types. These include the following: workplace aggression, which includes extremely violent actions, such as murder and assault, and more insidious behaviors, such as ignoring or withholding information (Baron & Neuman, 1996); workplace deviance, which includes wasting resources or putting workmates in harm’s way (Robinson & Bennett, 1995); counterproductive work behavior (Fox et al., 2001); organizational retaliatory behavior, which is retaliation for being treated unfairly (Skarlicki & Folger, 1997); and workplace incivility, behavior that merely lacks interpersonal consideration (Andersson & Pearson, 1999).

In Japan, unlike in the West, there is more disrespectful behavior and verbal violence than aggressive behavior like murder and assault. Aggressive behavior has been considered a human relations problem and has been treated as behavior that violates company rules, common sense, and moral standards in general society (Chae & Fukuhara, 2011). For instance, organizational retaliatory behavior (Tanaka, 2001), organizational malfunction behavior (Tanaka, 2002), and organizational dysfunctional behavior (Semba & Haraguchi, 2014; Tanaka, 2006, 2007; Tanaka & Toshima, 2003, 2005) fall into this category. Among them, this study examines “voluntary and intentional behaviors of workers that directly or indirectly have negative consequences for individuals or groups within the organization or the organization itself” (Semba & Haraguchi, 2014) as organizational dysfunctional behavior.

Japanese people tend to view the self as an entity in connection with others; they have a mutually cooperative view of the self that emphasizes adapting to the group and taking desirable actions and are concerned about how others evaluate their actions and choices (Fukuzawa et al., 2021). Therefore, when working in an organization, they are often conscious of how they are viewed by those around them. When working, maintaining a high sense of self-evaluation can be challenging. Often, individuals may face a crisis when their self-evaluation is threatened by making a mistake at work or being scolded by superiors. One theory that explains the motivation behind maintaining and enhancing self-evaluation is the sociometer theory (Leary & Baumeister, 2000), which posits that self-evaluation serves as a tool for measuring one’s acceptance or exclusion from others. Consequently, the feelings of individuals are likely influenced by their perceptions of how others evaluate them (Enomoto et al., 2001). In addition, several studies have shown that experiencing rejection from others increases aggression (Leary et al., 2006). One of the models that describes how individuals regulate and enhance their self-evaluation, while also choosing their behavior, is the self-oriented threat model (Baumeister et al., 1996; Baumeister & Boden, 1998).

Tanaka (2008) stated that the individual determinant of organizational dysfunctional behavior with the highest explanatory power is narcissism. Narcissism is a mentality that seeks to maintain a positive sense of self-evaluation. Other representative models that address narcissism and aggressive behavior include the social cognitive self-regulation model of narcissism (Rhodewalt, 2001), which does not depict the process by which an individual chooses aggressive behavior. On the other hand, the threatened egotism model depicts the process by which workers adjust their self-evaluations and choose their behaviors, thus allowing a detailed discussion of interventions. Therefore, the latter model was adopted and examined in this study. In the threatened egotism model, when individuals receive negative external evaluations, they perceive a crisis of self-devaluation (threatened egotism) due to a discrepancy with self-evaluation. If they then do not accept others’ evaluations, they are likely to maintain their self-evaluation, develop negative feelings toward others who have negatively evaluated them, and choose to engage in aggressive behavior.

On the other hand, the threatened egotism model also shows a process whereby the acceptance of others’ evaluations leads to a decrease in self-esteem, negative feelings toward oneself, and withdrawal from interpersonal relationships. Semba (2016b) conducted semi-structured interviews with 17 workers who engaged in aggressive behavior that interfered with organizational functioning. This study supported the relevance of the threatened egotism model to Japanese workers. Following this, a quantitative survey of workers was carried out, showing that these workers opted for aggressive behaviors in the workplace, which intensified their interpersonal phobia (Semba, 2016a, 2018). Furthermore, Semba (2019b) examined whether organizational dysfunctional behavior was regulated by individual attributes and suggested that it was not regulated by age. Semba (2021) also found a process where workers who feel rejected by others and perceive threatened egotism may behave aggressively when their expectations are not met. If these individuals have a strong ideal of how they “should be” toward others, they may lash out. On the other hand, if they have a strong ideal of how they “should be” toward themselves, they blame themselves for not meeting the expectations of others and withdraw from interpersonal relationships. Thus, although the threatened egotism model can explain how individuals choose aggressive behavior or intensify anthropophobia and withdraw from interpersonal relationships, specific measures to manage these behaviors have not been examined.

In recent years, some Japanese organizations have implemented anger management programs to manage behaviors such as aggression and withdrawal from interpersonal relationships (Japan Institute for Women’s Empowerment and Diversity Management, 2017). Anger management is defined as “a structured treatment to foster self-regulation of anger and aggressive behavior” (Novaco, 1975). In anger management group programs, the following skills are emphasized: (1) understanding the emotions of one’s self and others correctly through roleplaying and other activities, (2) objectively observing reasons for feeling anger and understanding personal anger patterns, and (3) learning coping skills for feelings of anger (Gulbenkoglu & Hagiliassis, 2006). Anger management has proven its usefulness to adults in the judiciary domain. For example, Dowden et al. (1999) divided incarcerated male federal offenders into a treated group, which received the institutional anger management program, and a comparison group and compared their recidivism rates after release. The results showed a significant reduction in the rates of nonviolent recidivism among the high-recidivism risk group that received treatment. In addition, police officers who underwent anger management training tended to make fewer arrests involving excessive use of force than those who did not receive such training (Abernethy & Cox, 1994).

From a psychological aspect, it has also been shown that failure to properly address feelings of anger can have negative effects on mental health, such as worsening depression and depression (Krakowski & Nolan, 2017). Participating in an anger management program can significantly help individuals deal with anger appropriately and maintain their mental health. A study conducted among university students in Japan indicated a decreasing tendency in both distrust toward others and aggression after attending an anger management program, comparing their attitudes before and after the experience (Kawamura & Kagawa, 2021). Steffen (2000) also examined the effects of anger management on middle-aged caregivers caring for patients with dementia and found lower levels of anger and depression and higher ratings of caregiving self-efficacy.

These previous studies have suggested that anger management attendance may lead to a reduction in aggressive behavior and interpersonal withdrawal. However, few studies have actually examined the effectiveness of anger management in management organizations, and it is unclear what effect it has on the problematic behavior of workers in organizations. Therefore, this study focused on interpersonal withdrawal and organizational dysfunctional behaviors, which are two problematic types of behaviors in Japan, and attempted to formulate the following two hypotheses and to test them with an intervention design (a pre–post-test without a control group).

**Hypothesis 1.**  *Anger management reduces interpersonal withdrawal.*

**Hypothesis 2.**  *Anger management reduces organizational dysfunctional behavior.*

## 2. Materials and Methods

### 2.1. Research Method

From June to November 2021, applicants for a free anger management program were recruited via member companies of the Association of Community-based Services in the Shikoku region of Japan, and the program and questionnaires (before the first session and after the third session of the program) were administered to the 92 workers who applied. Originally, I had planned to set up a control group, but the serious spread of COVID-19 in Japan made it difficult to gather as many survey subjects as I had planned. Therefore, I had no choice but to abandon setting up a control group.

### 2.2. Ethical Considerations

This study was approved by the Ethics Review Committee of the author’s affiliation (Reference Number 21-09) and conducted in accordance with general ethical guidelines in psychology. The participants were given a written and oral explanation of the purpose and procedures of the study. At the time, they were informed that they were free to (1) participate in the study; (2) withdraw from the study anytime, even after submitting the consent form; (3) delete their answers to the questionnaires; and (4) ask for clarification of any unclear points about the study. After the explanation, they were asked to sign a consent form, and all of the participants agreed and signed the form.

### 2.3. Anger Management Program

The anger management program consisted of three sessions, each consisting of a 90 min lecture and a 30 min work session, in which individuals worked on tasks and shared the results with their group (Table 1). The first session dealt with “Impulse Control” (the suppression of reflexive behavior, scoring anger, and becoming aware of core beliefs), the second with “Thought Control” and “Behavior Control” (the explanation of anger mechanisms, reviewing the meaning of events, and expanding/fixing/transmitting the range of tolerance), and the third with “Knowing the Characteristics of One’s Anger” and “Review” (the characteristics of one’s anger and a review of the program).

### 2.4. Survey Items

The survey items consisted of three parts: a face sheet, the Sociability Scale, and the Organizational Dysfunctional Behavior Scale.

#### 2.4.1. Face Sheet

The face sheet included an explanation of the purpose of the survey and the protection of privacy, along with questions on sex and age.

#### 2.4.2. Sociability Scale

This scale measures awareness of not being good at interpersonal relationships. It is based on the Interpersonal Awareness Scale for the Workplace (Hyugano & Oguchi, 2002), which measures the awareness of not being good at interpersonal relationships with specific people in occupational situations and was modified to measure the awareness of not being good at the interpersonal relationship itself without being limited to specific people (Hyugano, 2010; Oguchi et al., 2005).

It consists of two subscales: “Troublesomeness” and “Apprehension”. The participants were asked to reflect on their behavior in the workplace and to answer whether their behavior corresponded to the behavior described in the questionnaire using a five-point scale: “5—Strongly corresponded”, “4—Somewhat corresponded”, “3—Neither corresponded nor not corresponded”, “2—Somewhat not corresponded”, and “1—Strongly not corresponded”. The higher the score, the more likely the participant is to be concerned about their relationships with others.

The value of Cronbach’s alpha for the Sociability Scale was 0.75 before and 0.82 after the anger management program; for “Troublesomeness”, the value was 0.80 before and 0.89 after the program; and for “Apprehension”, the value was 0.66 both before and after the program.

#### 2.4.3. Organizational Dysfunctional Behavior Scale

This scale measures the frequency of behaviors that interfere with organizational functioning; it consists of three subscales: “Aggressive Assertiveness”, “Criticism of Others”, and “Rebellious Attitude” (Semba & Haraguchi, 2014). The participants were asked to reflect on their behavior in the workplace and to answer whether their behavior corresponded to the behavior described in the questionnaire using the following five-point scale: “5—Strongly corresponded”, “4—Somewhat corresponded”, “3—Neither corresponded nor not corresponded”, “2—Somewhat not corresponded”, and “1—Strongly not corresponded”. Higher scores indicate a greater tendency to choose organizational dysfunctional behavior.

The value of Cronbach’s alpha for the overall Organizational Dysfunctional Behavior Scale was 0.88 before and 0.87 after the program. The value for the subscale “Aggressive Assertiveness” was 0.77 before and 0.84 after the program; for “Criticism of Others”, the value was 0.76 before and 0.64 after the program; and for “Rebellious Attitude”, the value was 0.83 before and 0.76 after the program.

### 2.5. Flow of the Study

After conducting the pre-questionnaire survey (face sheet, Sociability Scale, and Organizational Dysfunctional Behavior Scale), the anger management program was conducted by a lecturer with a qualification as an Anger Management Training Professional^®^ certified by the Japanese Anger Management Association, followed by a post-questionnaire survey (Sociability Scale and Organizational Dysfunctional Behavior Scale) a month later.

### 2.6. Method of Analysis

#### 2.6.1. Analysis 1: Pre- and Post-Comparison of the Sociability Scale and the Organizational Dysfunctional Behavior Scale

Because the scores of the Sociability Scale (overall and subscales) and the Organizational Dysfunctional Behavior Scale (overall and subscales) did not follow a normal distribution, changes in the pre- and post-scores were not analyzed with the *t*-test, which assumes a normal distribution, but with the Wilcoxon signed-rank test, which does not.

#### 2.6.2. Analysis 2: Pre- and Post-Comparison of the Sociability Scale and the Organizational Dysfunctional Behavior Scale by Sex and Age

The same analysis as in Analysis 1 was conducted by sex and age (split into two by the median).

HAD ver.17.0 (Shimizu, 2016) was used for statistical analysis, and the significance level was set at 5%.

## 3. Results

All 92 participants submitted responses (100.0% response rate). Of them, 28 (30.4%) were men and 64 (69.6%) were women, with a mean age of 45.2 ± 11.3 years (40.2 ± 10.8 years for men and 47.3 ± 10.9 years for women).

### 3.1. Analysis 1: Pre- and Post-Comparison of the Sociability Scale and the Organizational Dysfunctional Behavior Scale

Regarding the Sociability Scale (overall and subscales), the “Troublesomeness” median remained unchanged at 2.2 from the pre- to post-comparison (*p* = 0.210). “Apprehension” decreased from 3.5 to 3.3, but no significant change was indicated (*p =* 0.805). The overall scale remained unchanged at 2.7 (*p =* 0.332) (Table 2).

Regarding the Organizational Dysfunctional Behavior Scale, the “Aggressive Assertiveness” and “Rebellious Attitude” medians remained unchanged at 2.0 and 2.3, respectively (*p =* 0.478, *p =* 0.528), and the overall scale changed from 2.5 to 2.3, but not significantly (*p =* 0.054). Only “Criticism of Others” significantly decreased from 3.2 to 3.0 (*p =* 0.011) (Table 3).

### 3.2. Analysis 2: Pre- and Post-Comparison of the Sociability Scale and the Organizational Dysfunctional Behavior Scale by Sex and Age

Similar analyses by sex and age revealed no significant changes in the median scores of the Sociability Scale (overall and subscales). On the other hand, regarding the Organizational Dysfunctional Behavior Scale, for men, “Aggressive Assertiveness” decreased from 2.0 to 1.8 but not significantly (*p =* 0.500). “Criticism of Others” and “Rebellious Attitude” remained unchanged at 2.7 and 2.3, respectively (*p =* 0.794, 0.712). The overall scale decreased from 2.3 to 2.1 but not significantly (*p =* 0.332) (Table 4).

For women, the median “Aggressive Assertiveness” score decreased from 2.3 to 2.0, but no significant change was recognized (*p =* 0.739). “Rebellious Attitude” remained unchanged at 2.3 (*p =* 0.553), and although the overall scale decreased from 2.6 to 2.4, no significant change was recognized (*p =* 0.095). Only “Criticism of Others” decreased significantly from 3.3 to 3.0 (*p =* 0.004) (Table 5).

Furthermore, for workers 46 years old or older, the median “Criticism of Others” score decreased from 3.3 to 3.0, but no significant change was recognized (*p =* 0.161). “Aggressive Assertiveness”, “Rebellious Attitude”, and the overall scale remained unchanged at 2.3, 2.3, and 2.6, respectively (*p =* 0.437, 0.634, and 0.232) (Table 6).

For those under 46 years old, “Aggressive Assertiveness” decreased from 2.0 to 1.8 but not significantly (*p =* 0.886). “Rebellious Attitude” remained unchanged at 2.0 (*p =* 0.690), and the overall scale decreased from 2.3 to 2.1 but not significantly (*p =* 0.131). Only “Criticism of Others” showed a significant change from 3.0 to 2.7 (*p =* 0.029) (Table 7).

## 4. Discussion

This study aimed to examine the effects of anger management on the interpersonal withdrawal and organizational dysfunctional behavior of workers in organizations. In order to achieve this objective, Hypotheses 1, “Anger management reduces interpersonal withdrawal”, and Hypothesis 2, “Anger management reduces organizational dysfunctional behavior”, were formulated and tested using a questionnaire survey.

First, regarding Hypothesis 1, “Anger management reduces interpersonal withdrawal”, Kawamura and Kagawa (2021) suggested that anger management was effective in reducing distrust of others among college students. However, the results of this study do not support this finding. One possible reason for this may be that this study targeted workers of a wide range of ages, while Kawamura and Kagawa (2021) targeted a specific age group: college students. Future analyses should attempt to analyze by age group. In addition, during the survey, Japan was amid the COVID-19 pandemic, and many Japanese organizations introduced teleworking and staggered work hours. Thus, the survey participants were forced to keep a certain distance from interpersonal relationships when the survey was conducted. In other words, they had withdrawn from interpersonal relationships from the beginning of the survey, which may have made the changes insignificant.

Second, regarding Hypothesis 2, “Anger management reduces organizational dysfunctional behavior”, the result that anger management is effective in reducing “Criticism of Others” is consistent with that of the study by Dowden et al. (1999), which suggested that anger management is effective in reducing nonviolent recidivism, as well as the study by Kawamura and Kagawa (2021), which suggested a reduction in aggression among anger management program participants.

In particular, “Criticism of Others” decreased significantly, but only for women and workers under 46 years of age due to attending the program.

The reason why no significant results were obtained for men may be that in modern male roles, interpersonal relationship skills are emphasized, and friendliness and intimacy are encouraged (Suzuki, 1994). In particular, because Japanese men in recent years have tended to strongly identify generosity with masculinity (Oishi & Kitakata, 2013), the scores before taking the anger management course were low, and as a result, the change may not have been significant. On the other hand, the reason for the significant decrease for women may be that the prior score for “Criticism of Others” was higher for women (3.3 for women and 2.7 for men), suggesting that there was more room for the decrease. Moreover, in Western countries, the self is seen as a unique entity separate from others, while in Asia, including Japan, the self is generally seen as part of a mutually connected network of human relationships (Markus & Kitayama, 1991). This suggests that the results of this study may have been significantly influenced by the cultural background of Japan.

Next, the finding that only workers under 46 years old experienced a significant decrease can be explained as follows: Because many workers under 46 years of age hold low positions in the workplace in Japan, it is assumed that they are less able to make decisions on their own and often have to conform to their superiors and seniors, even if their ideas are different from their own. According to Semba (2021), when workers’ ideas are rejected, and they follow the decisions of their superiors and seniors but do not agree with them, the image of their “ideal” superiors and seniors is betrayed. Therefore, when the matter decided by the supervisor or senior does not go well, they criticize their supervisors or seniors out of a self-defensive mentality to avoid responsibility. However, it is believed that attending the program reduced their criticism of others as they became aware of their self-defensive mental functioning. For these reasons, anger management may have controlled criticism of others among workers under 46 years old.

In Semba (2019b), age did not regulate organizational dysfunctional behavior. However, this study suggested that workers under 46 who attended the program were less likely to criticize others. Interestingly, age may be a determinant after workers attend anger management programs.

In contrast to the conventional studies on aggressive behavior in Japanese organizations, which have focused on the management of subordinates by supervisors, this study is unique and creative in that it introduces a new perspective of management by the workers themselves. Studies in this area have stalled because the behavior is not socially desirable, and the survey itself has been considered difficult. In a study of antisocial behavior in organizations, Tanaka (2008) found that personal factors have high explanatory power as determinants. Subsequently, a series of studies by Semba (2016b, 2016a, 2018, 2019a, 2021) focused on workers’ personal factors and examined aggressive behavior using the threatened egotism model. The results revealed that supervisor support reduced antisocial behavior in the organization. However, Semba’s series of studies was limited, as it relied on the supervisor’s management ability because it assumed that the supervisor would manage their subordinates. To overcome this limitation, this study attempted to help individuals manage their anger in the organization by attending an existing anger management program, focusing on anger, one of the most common negative emotions that can lead to aggressive behavior.

The academic contribution of this study is threefold. First, it made a novel contribution by examining the effects of anger management on Japanese general workers from a quantitative perspective. As far as I know, most practical studies on anger management in Japan have focused on children or students. For instance, Omori (2023) examined the effects of anger management on junior high school students using a program for children with developmental disability tendencies. Yajima and Oda (2021) examined the effects on the mental health of university students. Some other studies provided practical reports targeting children without sufficient verification of the effects of anger management (Enta et al., 2017; Honda & Takano, 2014; Ishiba, 2019; Isshi, 2016). On the other hand, the study on anger management targeting Japanese workers is limited to the study by Kobayashi (2023), which developed an anger management program to prevent power harassment. In this context, it is significant that this study examined the anger management effect for general workers in Japan.

Second, it contributes to the refinement of the threatened egotism model. The model can explain how individuals choose to act aggressively or withdraw from interpersonal relationships. In addition, this study suggests that anger management that enables individuals to manage their emotions and choose behaviors other than these types of problematic behaviors is a significant step toward refining the threatened egotism model.

Since 2000, problematic behaviors of workers in Japanese organizations, including “bad-mouthing”, “not listening to others’ opinions and imposing one’s own”, and “rebelling against superiors” have been reported (Tanaka & Toshima, 2003; Semba, 2016a), and studies contributing to their prevention have been started. However, most studies have been limited to discussions of determinants (Tanaka, 2008) and scale development (Semba & Haraguchi, 2014), hindering detailed discussions that would contribute to the prevention of problematic behaviors. The only exception is Semba’s series of studies (Semba, 2016a, 2016b, 2018, 2021, 2024), which examined the process of workers choosing these problematic behaviors, but not without limitations.

For example, Semba (2016b) conducted a quantitative study based on the threatened egotism model and suggested that perceived support from supervisors may reduce subordinates’ organizational dysfunctional behavior. However, the results were largely dependent on the support of supervisors and the model has not yet been refined. Semba (2016a, 2024) also explored the possibility of refining the model, but both surveys were interviews with workers, which limited the generalizability of the results. Significantly, this study paved the way for refinement of the threatened egotism model by showing the possibility of reducing organizational dysfunctional behavior by workers’ own efforts through a quantitative study.

Third, this study found that workers under the age of 46 who attended an anger management program were less likely to criticize others. It is academically significant that age may be a determinant of behavior after taking the program.

The practical contribution of this study is that it expands the area of application of anger management, which has been conducted primarily in education in healthcare and correctional facilities, and this shows its potential for application in education and guidance in management organizations. Although the expectation of reducing harassment through anger management has grown in Japanese management organizations, its effectiveness has not been measured. Regarding this issue, this study showed that attending an anger management program decreased “Criticism of Others” among women and workers under the age of 46. It is significant that anger management can help smooth communication among women and young people in the organization and, consequently, can be used to reduce harassment.

There are three limitations in this study. The first is the limitation of the sample. The participants were recruited from April to May 2021, when semi-emergency COVID-19 measures were imposed by the Japanese government. Thus, it was difficult to obtain cooperation from companies and organizations concerned about COVID-19 infection, and I could not gather as many participants as I had expected. This resulted in a much smaller sample size than the 200 originally planned for the survey, and I had to abandon the comparative study with a control group. Moreover, the participants in this study were predominantly women rather than men. It will be necessary in the future to increase the overall sample size and ensure a nearly equal number of men and women in the sample to increase the reliability of the results.

The second is the variation in the content of the anger management program. In this study, I employed the programs offered by the largest number of companies in Japan to conduct the survey. However, it is undeniable that the survey results may vary significantly depending on the content of the training programs. Therefore, in the future, it is necessary to address program variations and to try to gain a more comprehensive understanding of the results.

The third is a difference in the motivation for attending the program. This study recruited participants for an anger management program via member companies of a cooperating organization. While some participants decided to attend the program of their own volition, others were urged to attend it by their supervisors or managers. The latter may not have responded truthfully to the questionnaire, considering social desirability. It is undeniable that this difference in motivation may have influenced the results of the study. In the future, it will be necessary to analyze the results according to the motivation for attending the program.

It is recommended that future research be conducted to examine what type of organizational dysfunctional behavior workers tend to select by their attributes. Then, if effective programs can be developed to match the tendency of each attribute to select organizational dysfunctional behaviors, those behaviors of employees will be reduced.

## 5. Conclusions

A questionnaire survey conducted among participants of an anger management program revealed the following results: Hypothesis 1, which stated that “Anger management reduces interpersonal withdrawal”, was found to be false; and Hypothesis 2, which stated that “Anger management reduces organizational dysfunctional behavior”, was found to be true for the criticism of others among women and young workers.

## Figures and Tables

**Table 1:** Overview of the anger management program.

Session ^1^ Date	Themes	Contents
1 20 November	Impulse Control	Suppression of Reflexive Behavior Think of a way to persevere through the 6 s peak of anger and present it to the group. Scoring Anger Score one’s angry events and be objective about one’s anger. Becoming Aware of Core Beliefs Know “should” in oneself.
2 8 January	Thought Control Behavior Control	Explanation of Anger Mechanisms Learn the mechanism of anger and understand the primary emotion that is the source of anger. Reviewing the Meaning of Events Recall one’s angry events and categorize them into “things one can change”, “things one can’t change”, “important”, and “unimportant”. Expanding/Fixing/Transmitting the Range of Tolerance Classify one’s angry events into “forgivable”, “tolerable”, and “unforgivable”. Aim to expand and fix the range of “tolerable”, consider how to communicate one’s “tolerable” boundary to others.
3 5 March	Knowing the Characteristics of One’s Anger Review	Characteristics of One’s Anger Learn about the “intensity”, “frequency”, “aggression”, and “persistence” of one’s anger. Review of the Program Reflect on the three sessions and present what one has noticed and thought about.

^1^ Each session consisted of 90 min of lecture and 30 min of work, comprising individual and group work. In the individual work portion, the participants reflected on their personal experiences related to the lecture topics and filled out a sheet. In the group work portion, each member shared their personal experience based on the sheet.

**Table 2:** Prior–post comparison of the Sociability Scale.

	Prior ^1^	Post ^1^	Z-Value ^2^	*p*-Value ^2^
Troublesomeness	2.2	2.2	−1.253 ^3^	0.210
Apprehension	3.5	3.3	0.246 ^4^	0.805
Overall Scale	2.7	2.7	−0.969 ^3^	0.332

^1^ Median; ^2^ Wilcoxon signed-rank test with continuity correction; ^3^ based on negative ranks; ^4^ based on positive ranks.

**Table 3:** Prior–post comparison of the Organizational Dysfunctional Behavior Scale.

	Prior ^1^	Post ^1^	Z-Value ^2^	*p*-Value ^2^
Aggressive Assertiveness	2.0	2.0	−0.709 ^3^	0.478
Criticism of Others	3.2	3.0	2.558 ^3^	0.011
Rebellious Attitude	2.3	2.3	0.632 ^3^	0.528
Overall Scale	2.5	2.3	1.925 ^3^	0.054

^1^ Median; ^2^ Wilcoxon signed-rank test with continuity correction; ^3^ based on positive ranks.

**Table 4:** Prior–post comparison of the Organizational Dysfunctional Behavior Scale (men).

	Prior ^1^	Post ^1^	Z-Value ^2^	*p*-Value ^2^
Aggressive Assertiveness	2.0	1.8	0.675 ^3^	0.500
Criticism of Others	2.7	2.7	0.261 ^3^	0.794
Rebellious Attitude	2.3	2.3	0.369 ^3^	0.712
Overall Scale	2.3	2.1	0.971 ^3^	0.332

^1^ Median; ^2^ Wilcoxon signed-rank test with continuity correction; ^3^ based on positive ranks.

**Table 5:** Prior–post comparison of the Organizational Dysfunctional Behavior Scale (women).

	Prior ^1^	Post ^1^	Z-Value ^2^	*p*-Value ^2^
Aggressive Assertiveness	2.3	2.0	−0.333 ^3^	0.739
Criticism of Others	3.3	3.0	2.876 ^3^	0.004
Rebellious Attitude	2.3	2.3	0.594 ^3^	0.553
Overall Scale	2.6	2.4	1.668 ^3^	0.095

^1^ Median; ^2^ Wilcoxon signed-rank test with continuity correction; ^3^ based on positive ranks.

**Table 6:** Prior–post comparison of the Organizational Dysfunctional Behavior Scale (46 years old or older).

	Prior ^1^	Post ^1^	Z-Value ^2^	*p*-Value ^2^
Aggressive Assertiveness	2.3	2.3	0.778 ^3^	0.437
Criticism of Others	3.3	3.0	1.400 ^3^	0.161
Rebellious Attitude	2.3	2.3	0.476 ^3^	0.634
Overall Scale	2.6	2.6	1.196 ^3^	0.232

^1^ Median; ^2^ Wilcoxon signed-rank test with continuity correction; ^3^ based on positive ranks.

**Table 7:** Prior–post comparison of the Organizational Dysfunctional Behavior Scale (under 46 years old).

	Prior ^1^	Post ^1^	Z-Value ^2^	*p*-Value ^2^
Aggressive Assertiveness	2.0	1.8	−0.143 ^3^	0.886
Criticism of Others	3.0	2.7	2.188 ^3^	0.029
Rebellious Attitude	2.0	2.0	0.398 ^3^	0.690
Overall Scale	2.3	2.1	1.508 ^3^	0.131

^1^ Median; ^2^ Wilcoxon signed-rank test with continuity correction; ^3^ based on positive ranks.

## Data Availability

Data supporting the findings of this study are available from the author upon request.
